# The Role of Baseline PET/CT Parameters in Predicting Treatment Response in Patients with Locally Advanced Rectal Cancer Undergoing Total Neoadjuvant Therapy

**DOI:** 10.3390/medicina61081449

**Published:** 2025-08-12

**Authors:** Ali Kaan Güren, Zilan Başkan, Zeynep Ceren Balaban Genç, Tuğçe Bulun Akyol, Erkam Kocaaslan, Yeşim Ağyol, Pınar Erel, Burak Paçacı, Mustafa Alperen Tunç, Ahmet Demirel, Nargiz Majidova, Nadiye Sever, Abdussamet Çelebi, Selver Işık, Murat Sarı, Osman Köstek, Salih Özgüven, Hilal Alkış, Mustafa Adli, İbrahim Vedat Bayoğlu

**Affiliations:** 1 Division of Medical Oncology, Department of Internal Medicine, Marmara University School of Medicine, Istanbul 34854, Turkey; erkamkocaaslan@gmail.com (E.K.); yesimagyol@gmail.com (Y.A.); pnarerell@gmail.com (P.E.); drpacaci@gmail.com (B.P.); m.alperen.tunc@gmail.com (M.A.T.); drahmetdemirel23@gmail.com (A.D.); dr.nadya@hotmail.com (N.S.); abdussametcelebi@gmail.com (A.Ç.); dr-selver83@hotmail.com (S.I.); drmuratsari@gmail.com (M.S.); osmankostek@yahoo.com (O.K.); dr.vebay@gmail.com (İ.V.B.); 2 Department of Radiation Oncology, Marmara University School of Medicine, Istanbul 34854, Turkey; zilanbaskan@hotmail.com (Z.B.); hilal.alkis@marmara.edu.tr (H.A.); mustafa.adli@marmara.edu.tr (M.A.); 3 Department of Nuclear Medicine, Marmara University School of Medicine, Istanbul 34854, Turkey; zcbalabangenc@gmail.com (Z.C.B.G.); drsozg@gmail.com (S.Ö.); 4 Department of Internal Medicine, Marmara University School of Medicine, Istanbul 34854, Turkey; tugcebulunmd@gmail.com; 5 Department of Medical Oncology, VM Medical Park Maltepe Hospital, Istanbul 34846, Turkey; nergiz.mecidova1991@gmail.com

**Keywords:** locally advanced rectal cancer, total neoadjuvant therapy, total lesion glycolysis, metabolic tumor volume, complete response

## Abstract

*Background and Objectives:* Total neoadjuvant therapy (TNT) for locally advanced rectal cancer (LARC) offers significant advantages in terms of pathologic response and long-term survival; however, it is still unclear which patients will benefit the most from this treatment. This study aims to investigate the role of metabolic parameters on pretreatment positron emission tomography–computed tomography (PET/CT) images in predicting treatment response after TNT. *Materials and Methods:* The research was conducted using a single-center, retrospective design. Patients treated with total neoadjuvant therapy are included if they have locally advanced rectal cancer (cT3/T4-N0 or cTany-N1/N2). The patient group was categorized into two groups: CR and non-CR. Clinicopathologic features, PET/CT parameters, CA19-9, and CEA values were compared between these two groups. *Results:* In total, 52 patients were included. The CR group had 21 patients, and the non-CR group had 31 patients. The analysis demonstrated that the CR group had significantly lower metabolic tumor volume (MTV) and total lesion glycolysis (TLG) than the non-CR group (*p* = 0.022 vs. *p* = 0.003, *p* < 0.05). Also, CA19-9 values were lower than the non-CR group, and this difference was statistically remarkable (*p* = 0.40, *p* < 0.05). *Conclusions:* MTV and TLG parameters in PET/CT for pretreatment staging and pretreatment blood CA 19-9 levels are prognostic factors for predicting treatment response, and they may play a crucial role in choosing treatment. Comprehensive research is warranted on this subject with a larger patient population.

## 1. Introduction

For many years, neoadjuvant long-course chemoradiotherapy (LCRT) or short-course radiotherapy (SCRT), both followed by total mesorectal excision and adjuvant chemotherapy, were standard therapy for locally advanced rectal cancer (LARC) [1,2]. In recent years, the pathological complete response (pCR), disease-free survival (DFS), and overall survival (OS) rates obtained in the Polish II, RAPIDO, STELLAR, and UNICANCER-PRODİGE 23 trials, which investigated the efficacy of total neoadjuvant therapy (TNT), showed that TNT is a new and significant treatment option in LARC treatment [3,4,5,6].

All studies concluded that TNT application had a higher rate of pathological complete response (pCR) than standard neoadjuvant chemoradiotherapy (nCRT). However, these studies had differences in the designs of these trials, and the treatment agents at the endpoints of trials are controversial. Meta-analyses of studies, including clinical trials and other real-life data, showed significant distant recurrence, DFS, and OS contributions [7]. Additionally, TNT provided significant benefits, such as increasing the rate of sphincter-sparing surgery by decreasing tumor size. Meanwhile, the surgical complication rates are similar [8]. After the successful results, TNT has become the standard recommendation, especially with high-risk and lower rectum invasion LARC patients [9]. The increased clinical complete response (cCR) rates have expanded the potential patient population for nonoperative management strategies (NOM). The successful results of studies investigating the watch-and-wait (WW) protocol, particularly the OPRA study, demonstrated the success and feasibility of NOM strategies [10,11]. NOM seems a promising and important option for the future as patients continue their physiologic lives and survival times are at similar levels, although discussions continue for the NOM approach [12].

In recent years, studies have shown that positron emission tomography–computed tomography (PET/CT) parameters are related to treatment response in many cancers. Specifically, total lesion glycolysis (TLG) and metabolic tumor volume (MTV) are related to treatment response and tumor aggressivity [13,14]. Much research has been conducted to foresee TLG and MTV’s predictability of treatment response in LARC patients treated with standard nCRT. These studies have concluded that high TLG and MTV levels are negative cCR predictors [15,16].

It is still controversial who is eligible for the TNT approach after TNT has elevated cCR rates for the potential NOM patient numbers. Before treatment, the CR expected-patient group must be defined well. Based on this, we aim to reveal the relationship between cCR and pCR response with clinicopathological features and values such as mean standardized uptake value (SUVmean), maximum standardized uptake value (SUVmax), TLG, MTV, and mean standardized uptake value (SUVmean) in PET/CTs taken for pretreatment staging in patients treated with TNT with a prediagnosis of LARC.

## 2. Materials and Methods

### 2.1. Study Population and Data Collection

The research was conducted using a single-center, retrospective design. Between 01.06.2019 and 01.06.2024, patients diagnosed with rectal cancer by histopathologic examination who were in a locally advanced stage (cT3/T4-N0 or cTany-N1/N2) at the time of diagnosis were included in the study. The patients’ data were reviewed using patient files and the hospital’s electronic data system. For diagnostic staging, the American Joint Committee on Cancer (AJCC) tumor/node/metastasis (TNM) classification and staging system 8. Edition used. The LARC staging has been confirmed with digital rectal examination, pelvic magnetic resonance imaging (MRI), and PET/CT. All patients were treated with total neoadjuvant therapy.

Demographic and clinicopathologic characteristics, baseline carcinoembryonic antigen (CEA), carbohydrate antigen 19-9 (CA19-9) values, treatment regimens, treatment responses, and lesion and liver SUVmean, SUVmax, and lesion TLG and MTV values on pretreatment PET/CT were recorded. Performance scores were calculated using the Eastern Cooperative Oncology Group Performance Status (ECOG PS).

### 2.2. Treatment Protocols and 18F-FDG PET/CT Imaging and Analysis

Patients received LCRT or SCRT according to the TNT protocol. Patients who received LCRT were administered a total dose of 50.4–56 Gy to the primary tumor and 45–50.4 Gy to the regional lymph nodes, which include the presacral space, internal iliac nodes, obturator nodes, and ischiorectal fossa. This treatment was delivered in 25–28 fractions with concurrent capecitabine at 825 mg/m^2^ orally twice daily, five days a week, during the RT period. In contrast, patients receiving SCRT were administered a total of 25 Gy to the primary tumor and the regional lymph nodes in just five fractions. Patients who received LCRT received concurrent chemotherapy, followed by four to six cycles of chemotherapy. Patients who received SCRT received RT only, followed by four or six cycles of chemotherapy. CAPOX and FOLFOX4 were used as systemic chemotherapy regimens.

All 18F-FDG PET/CT scans were conducted using a Discovery ST PET/CT scanner (GE Healthcare, Milwaukee, WI, USA). A cuboid volume of interest (VOI) was defined to encompass the rectal cancer lesion, and the VOI boundaries were automatically delineated along the tumor uptake margins based on a predetermined SUV threshold using PET VCAR software (GE HealthCare, Chicago, IL, USA). The SUVmax was identified as the highest standardized uptake value within the VOI. The MTV was determined as the portion of the tumor exhibiting SUV values equal to or exceeding 40% of the SUVmax within the VOI. The SUVmean was calculated as the average SUV within the VOI. TLG was derived by multiplying the MTV by the SUVmean.

Measurements of tumor length in the vertical axis, SUVmax, SUVmean, MTV, and TLG were recorded before and after 18F-FDG PET/CT imaging. Additionally, SUVmax values of the liver parenchyma were assessed as a standard reference parameter. Two experienced nuclear medicine specialists carried out the final evaluation of CT, PET, and fused PET/CT images.

### 2.3. Study Design and Statistical Analysis

Treatment response was evaluated using DRE, rectosigmoidoscopy, biopsy, and pelvic MRI. Patients with no residual tumor on DRE, biopsy, and MRI were considered cCR. According to treatment response, patients were divided into two categories: CR and non-CR. Patients who relapsed within the first 6 months after the end of treatment were not considered cCR. Essential demographic characteristics such as age and gender, tumor size, localization (distance from the anal verge), clinical stage at diagnosis (cTxNx), pretreatment CEA, and CA19-9 values were compared between the groups. In addition, SUVmean, SUVmax, TLG, and MTV values on pretreatment PET/CT were compared between the groups.

SPSS version 22.0 (IBM Corp., Armonk, NY, USA) was used for all statistics. While evaluating the study data, the conformity of the parameters to normal distribution was evaluated using Kolmogorov–Smirnov and Shapiro–Wilk tests. In the analysis of continuous variables between two groups, the Independent Samples *t*-test was used if the data were normally distributed, and the Mann–Whitney U Test was used if the data were not normally distributed. The chi-square test was used to analyze categorical variables. Significance was evaluated at *p* < 0.05 level.

## 3. Results

### 3.1. Characteristics of Patients 

A total of 307 patients diagnosed with rectal cancer were scanned. The study included 52 patients who underwent TNT for LARC and had PET/CT for pretreatment staging. There were 21 patients in the CR group and 31 in the non-CR group. The median age of the patients ranged between 40 and 89 years. An amount of 35 (67.3%) patients were men, and 17 (32.6%) were women; 48 of 52 patients had an ECOG PS of 0 or 1. In 29 patients, the largest diameter of the tumor was 5 cm or less, while the largest diameter was larger than 5 cm in 23 patients. In 31 patients, the tumor was located in the distal, 14 in the middle, and 7 in the upper rectum. All 40 patients whose microsatellite status was reached were microsatellite stable (MSS). At the time of diagnosis, eight patients had cT2, 41 patients had cT3, three patients had cT4 disease, four patients had cN0, 37 had cN1, and 11 had cN2 disease. In total, five patients received short-course radiotherapy, while 47 received long-course radiotherapy. In addition, 49 patients were treated with the radiotherapy concurrent capecitabine followed by CAPOX, while three patients were treated with the radiotherapy concurrent 5-Fluorouracil infusion followed by the FOLFOX regimen. All patients received 4–6 cycles of CAPOX therapy. Patient characteristics and other findings are summarized in Table 1.

### 3.2. Pretreatment PET/CT Measurements and Treatment Response

The mean lesion SUVmean was 10.14 ± 5.55, SUVmax was 17.59 ± 9.51, lesion-to-liver SUVmean was 5.04 ± 2.48, and SUVmax was 6.46 ± 3.05 on pretreatment PET/CTs. The median MTV value was 19.72 ± 10.13 cm^3^, while the median TLG value was 190.76 ± 137.23. The findings and distribution according to the groups are summarized in Table 2.

**Figure 1:**
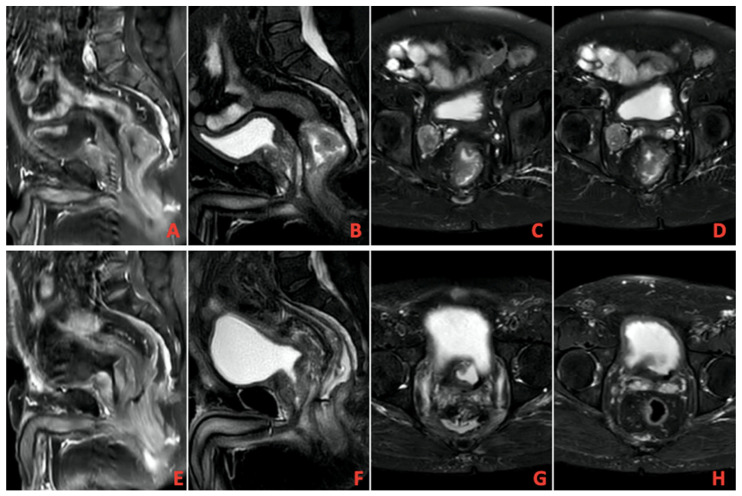
(**A**–**D**) Pretreatment imaging, (**E**–**H**) Posttreatment imaging on magnetic resonance imaging (MRI).

**Figure 2:**
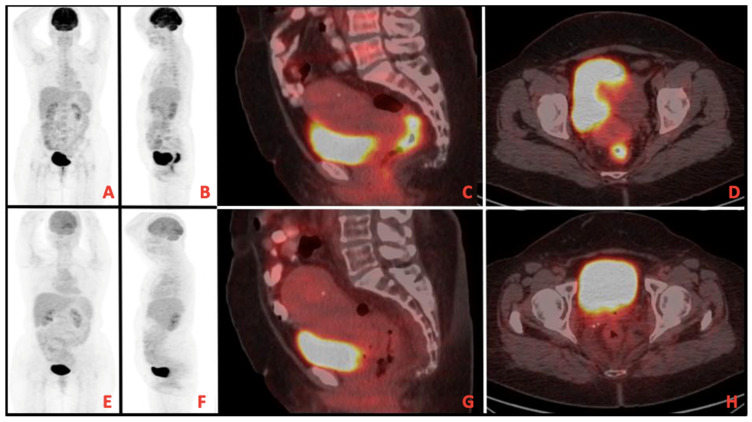
(**A**–**D**) Pretreatment imaging, (**E**–**H**) posttreatment imaging on positron emission tomography/computed tomography (PET/CT).

A total of 13 patients evaluated as cCR after TNT was completed were followed up with NOM. Figure 1 shows MRI images of a patient with complete response, and Figure 2 shows PET/CT images. It shows that 39 patients underwent surgery, and 8 of them had pCR. A total of 9 patients had ypT0 (one of them was ypT0N1), 1 had ypT1, 6 had ypT2, 21 had ypT3, and 1 had ypT4. In addition, 27 patients had no pathologically involved lymph nodes (ypN0), 9 had ypN1, and 2 had ypN2. These findings and tumor regression grading distribution are summarized in Table 3.

### 3.3. Comparison of CR and Non-CR Groups

The median age of the patients was 60.47 ± 11.95 years in the CR group and 62.93 ± 11.78 years in the non-CR group. The proportion of male patients was 71.4% in the CR group and 64.5% in the non-CR group. The mean diameter of the largest tumor was 5.69 ± 2.03 cm in the CR group and 6.14 ± 1.74 cm in the non-CR group. According to Distance from Anal Verge measurements, the median distance was 5.16 ± 3.32 cm in the CR group and 6.23 ± 4.03 cm in the non-CR group. When the CR and non-CR groups were analyzed according to the clinical stage distribution (cT and cN) before treatment, no significant difference was found between the two groups (*p* = 0.105 and *p* = 0.228, respectively).

Regarding PET/CT parameters, lesion-to-liver SUVmean values were 4.49 ± 2.19 in the CR group and 5.42 ± 2.62 in the non-CR group, and this difference was not statistically significant (*p* = 0.414). Lesion-to-liver SUVmax values were 5.59 ± 2.61 in the CR group and 7.05 ± 3.03 in the non-CR group, and the difference between the groups was not statistically significant (*p* = 0.355). MTV was 15.92 ± 6.52 cm^3^ in the CR group and 22.29 ± 11.37 cm^3^ in the non-CR group. This difference was statistically significant (*p* = 0.022). TLG values were 129.91 ± 60.77 SUV × cm^3^ in the CR group, and 231.98 ± 158.77 SUV × cm^3^ in the non-CR group, and the difference was significant (*p* = 0.003).

The median CEA levels were 4.2 (0–25.4) ng/mL in the CR group and 6.1 (0–87.5) ng/mL in the non-CR group, and this difference between the groups was insignificant (*p* = 0.077). CA19-9 levels were 65 (0–525) U/mL in the CR group and 117 (0–1358) U/mL in the non-CR group. This difference was statistically significant (*p* = 0.040). All these findings are summarized in Table 4.

## 4. Discussion

In TNT-treated LARC patients, there was a significant correlation between MTV and TLG values measured on pretreatment PET/CT and patients’ response to treatment. MTV and TLG values were statistically significantly lower in the pCR group compared to the non-PCR group. Considering the increasing use of TNT and NOM strategies, these pretreatment assessments may help clinicians predict the patient’s response to treatment and determine the treatment modalities chosen.

The relationship between standard nCRT, MTV, and TLG has been examined in previous years. Many studies have found that high pretreatment MTV and TLG values are associated with lower pathologic response rates and negative survival outcomes after nCRT [17,18]. Also, decreased MTV and TLG values have been reported to indicate a favorable response to treatment [19,20]. In our study, a statistically significant difference was detected between the MTV and TLG rates between the CR group and the non-CR group, and this result emphasizes the value of MTV and TLG while predicting treatment response. Some studies have shown a relationship between SUVmax values and complete response in patients receiving standard CRT [21,22]. However, our results showed no statistical difference between SUVmean and SUVmax between CR and non-CR groups. Studies involving more patients are needed to eliminate the contradictions in this regard.

Previous studies found a significant relationship between CA19-9 and CEA values measured before treatment in LARC patients in terms of treatment response, disease recurrence, and overall survival [23,24]. In our study, CA19-9 and CEA values were numerically higher in the non-PCR group compared to the CR response group. However, when comparing the CR and non-CR groups, this difference was statistically significant only between CA19-9 values. This result indicates that CA19-9 values measured before treatment may be predictive in predicting treatment response. Survival results are currently immature as median DFS and OS were not reached.

In previous studies, the CR rates obtained with TNT varied between 22.4% and 29.9% [25,26]. In our study, this rate was found to be 40.3%. The main reason was that 90.3% of our patients had received LCRT, and 63.8% had received 56 Gy. Plus, while 38.1% of our patients were pCR, 61.9% were cCR. Although the patients who were accepted as cCR were patients who did not relapse within 6 months, we think that the higher rate of cCR compared to pCR may have increased the total number of CRs.

While standard nCRT was used in the Polish II, RAPIDO, and STELLAR studies, LCRT was used in the UNICANCER-PRODIGO 23 study. In the preoperative period, FOLFIRINOX (folinic acid, 5-fluorouracil, irinotecan, oxaliplatin) was used in the UNICANCER-PRODIGO 23 study, FOLFOX4 (folinic acid, 5-fluorouracil, oxaliplatin), or CAPOX (capecitabine and oxaliplatin) in the RAPIDO study, FOLFOX4 in Polish II, and CAPOX in the STELLAR study [3,4,5,6]. Unlike other studies, the NOM approach was also used in patients with complete response in the STELLAR study [5]. Looking at 3-year DFS, an increase was observed in the TNT arm compared to standard CRT in the RAPIDO and UNICANCER-PRODİGE 23 trials. However, OS was similar in both groups in the RAPIDO study. In the STELLAR study, while DFS was similar between the groups, an increase in OS was observed in the TNT arm. Although the treatment modalities and survival analyses in these major studies differed, increased CR rates were obtained with the TNT approach compared to the standard CRT approach in all modalities applied [3,4,5,6]. However, there are still no precise data on which patient groups achieved higher CR rates in all these studies. In selecting the treatment modality to be applied in elderly, fragile patients with uncertain complete responses in whom surgical decision-making is complex, patient groups that will respond well to TNT should be better defined.

Regardless of whether standard neoadjuvant nCRT or TNT is used, the feasibility of NOM in patients who achieve complete or near-complete response has been a topic of ongoing debate for a long time [27,28,29]. Primarily, the OPRA study provides crucial information in this regard. The OPRA study applied the WW strategy to 74% of patients with a complete or nearly complete response, and tumor regrowth developed in 36%. When the operated patients in this group were compared with those initially treated with TME for non-CR, the 5-year DFS was 64% in both groups, and there was no difference between them [11]. One of the three patients who underwent WW recurred. From this point of view, we believe that the clinicopathologic features of the group with recurrence should be defined in more detail. Studies comparing TLG, MTV, and CA 19-9 levels with recurrence rate may help clinicians.

The limitations of our study were that it included a small group of patients, the follow-up period was short, and PET/CT was not performed for treatment response in every patient before and after TNT. Subgroup analyses could not be detailed due to the small number of patients. In addition, since TNT is a new treatment, data on recurrence rate, median DFS, and OS were inaccessible. Nevertheless, our study differs from other studies in that it focuses on a new approach, such as TNT, and, as far as we can see, is the first study on this subject.

## 5. Conclusions

Following the outstanding results of TNT, the number of patients achieving CR and, accordingly, the importance of NOM strategies are increasing. With the increase in the number of patients with CR and WW, the frequency of follow-up and the choice of diagnostic tools used in follow-up gain importance. In order to determine all these more clearly, patient groups and tumor characteristics need to be more accurately defined. Accordingly, measuring parameters such as MTV and TLG on PET/CT for preoperative staging are important prognostic factors in predicting treatment response. Low preoperative MTV and TLG values may be considered a predictive factor for complete response in patients who cannot be evaluated for response with rectosigmoidoscopy and pelvic MRI when deciding on NOM. Although the findings of our study reveal the relationship between treatment response and PET/CT parameters in the context of TNT an area that has not been previously explored, comprehensive studies with larger patient populations are warranted in this field.

## Figures and Tables

**Table 1:** Baseline demographic and clinicopathological characteristics of the patients and treatment modalities implemented.

	CR (n = 21)	Non-CR (n = 31)	Total (n = 52)
Age (min–max)	60 (41–82)	62 (40–89)	61 (40–89)
Gender (%)			
Male	15 (71.4)	20 (64.5)	35 (67.3)
Female	6 (28.5)	11 (35.4)	17 (32.6)
ECOG PS (%)			
0–1	20 (95.2)	28 (90.3)	48 (92.3)
2	1 (4.7)	3 (9.6)	4 (7.6)
Tumor size (%)			
≤5 cm	12 (57.1)	17 (54.8)	29 (55.7)
>5 cm	9 (42.8)	14 (45.1)	23 (44.2)
Distance from Anal Verge			
≤5 cm	12 (57.1)	19 (61.2)	31 (59.6)
5–10 cm	7 (33.3)	7 (22.6)	14 (26.9)
10–15 cm	2 (9.5)	5 (16.1)	7 (13.4)
Microsatellite status (%)			
MSS	16 (100)	24 (100)	40 (100)
MSI/H	0 (0)	0 (0)	0 (0)
Unknown	5	7	12
Clinical T Stage (%)			
T2	3 (14.2)	5 (16.1)	8 (15.4)
T3	17 (80.9)	24 (77.4)	41 (78.8)
T4	1 (4.7)	2 (6.4)	3 (5.7)
Clinical N Stage (%)			
N0	2 (9.5)	2 (6.4)	4 (7.6)
N1	15 (71.4)	22 (70.9)	37 (71.2)
N2	4 (19)	7 (22.5)	11 (21.1)
Radiotherapy			
Short-course	2 (9.5)	3 (9.6)	5 (9.7)
Long-course	19 (90.4)	28 (90.3)	47 (90.3)
Concurrent chemotherapy			
Capecitabine	20 (95.2)	29 (93.5)	49 (94.2)
5-Fluorouracil infusion	1 (4.7)	2 (6.4)	3 (5.7)
Chemotherapy regimens			
CAPOX	20 (95.2)	29 (93.5)	49 (94.2)
FOLFOX	1 (4.7)	2 (6.4)	3 (5.7)
Chemotherapy Cycles (Capox) (n = 49)			
4 cycles	7 (35)	11 (37.9)	18 (36.7)
5 cycles	5 (25)	5 (17.2)	10 (20.4)
6 cycles	8 (40)	13 (44.8)	21 (42.8)

CR: complete response; ECOG PS: the Eastern Cooperative Oncology Group performance score; MSS: microsatellite Stable; MSI/H: Microsatellite Instability-High; FOLFOX: Fluorouracil+Folinic acid+Oxaliplatin; Capox: Capecitabine+Oxaliplatin.

**Table 2:** Pretreatment PET/CT measurements, encompassing SUVmean, SUVmax, MTV, and TLG.

	CR (n = 21)	Non-CR (n = 31)	Total (n = 52)
	Mean ± SD	Mean ± SD	Mean ± SD
Lesion SUVmean	8.54 ± 4.94	11.22 ± 5.76	10.14 ± 5.55
Lesion SUVmax	14.86 ± 8.36	19.44 ± 9.93	17.59 ± 9.51
Lesion-to-liver SUVmean	4.49 ± 2.19	5.42 ± 2.62	5.04 ± 2.48
Lesion-to-liver SUVmax	5.59 ± 2.61	7.05 ± 3.03	6.46 ± 3.05
Metabolic Tumor Volume (MTV)	15.92 ± 6.52	22.29 ± 11.37	19.72 ± 10.13
Total Lesion Glycolysis (TLG)	129.91 ± 60.77	231.98 ± 158.77	190.76 ± 137.23

CR: complete response; SD: standard deviation; SUV: standardized uptake values.

**Table 3:** Treatment outcomes.

	Patients (%)
Surgery (n = 52)	
Yes	39 (75)
No	13 (25)
Complete Response (n = 21)	
Pathological CR	8 (38.1)
Clinical CR	13 (61.9)
Pathologic T category (n = 38)	
ypT0	9 (23.6)
ypT1	1 (2.6)
ypT2	6 (15.7)
ypT3	21 (55.2)
ypT4	1 (2.6)
Pathologic N category (n = 38)	
ypN0	27 (71)
ypN1	9 (23.6)
ypN2	2 (5.2)
Tumor regression grading (n = 38)	
0	8 (21)
1	12 (31.5)
2	10 (26.3)
3	8 (21)

CR: complete response.

**Table 4:** Comparison of patient characteristics and PET/CT parameters regarding CR and Non-CR groups.

	CR (n = 21)	Non-CR (n = 31)	*p* Value
	Mean ± SD	Mean ± SD	
Age (years)	60.47 ± 11.95	62.93 ± 11.78	0.911 *
Tumor size (cm)	5.69 ± 2.03	6.14 ± 1.74	0.472 *
Distance from Anal Verge (cm)	5.16 ± 3.32	6.23 ± 4.03	0.477 *
Lesion-to-liver SUVmean	4.49 ± 2.19	5.42 ± 2.62	0.414 *
Lesion-to-liver SUVmax	5.59 ± 2.61	7.05 ± 3.03	0.355 *
Metabolic Tumor Volume (cm^3^)	15.92 ± 6.52	22.29 ± 11.37	**0.022** *
Total Lesion Glycolysis (SUV × cm^3^)	129.91 ± 60.77	231.98 ± 158.77	**0.003** *
CEA (ng/mL) (median, min–max)	4.2 (0–25.4)	6.1 (0–87.5)	0.077 **
CA19-9 (U/mL) (median, min–max)	65 (0–525)	117 (0–1358)	**0.040** **
	n (%)	n (%)	*p* value
Gender			0.602 ***
Male	15 (71.4)	20 (64.5)	
Female	6 (28.5)	11 (35.4)	
Clinical T Stage			0.105 ***
T2	3 (14.2)	5 (16.1)	
T3	17 (80.9)	24 (77.4)	
T4	1 (4.7)	2 (6.4)	
Clinical N Stage			0.228 ***
N0	2 (9.5)	2 (6.4)	
N1	15 (71.4)	22 (70.9)	
N2	4 (19)	7 (22.5)	
Long-course Radiotherapy Dose (n = 47)			0.149 ***
56 Gy	14 (70)	16 (59.2)	
50 Gy	6 (30)	11 (40.7)	
Chemotherapy Cycles (Capox) (n = 49)			0.415 ***
4 cycles	7 (35)	11 (37.9)	
5 cycles	5 (25)	5 (17.2)	
6 cycles	8 (40)	13 (44.8)	

* Independent Samples test; ** Mann–Whitney U Test; *** chi-square test. CEA: carcinoembryonic antigen; CA19-9: carbohydrate antigen 19-9; SUV: standardized uptake values; Capox: Capecitabine+Oxaliplatin.

## Data Availability

The data supporting this study’s findings are not publicly available due to privacy reasons but are available from the corresponding author.
